# Cytological Clues to Pharyngoesophageal Diverticula Mimicking Thyroid Nodules

**DOI:** 10.1002/dc.70100

**Published:** 2026-02-11

**Authors:** Yu‐Wei Lin, Tzu‐Hang Kao, Tsung‐Lun Lee, Jen‐Fan Hang

**Affiliations:** ^1^ Department of Pathology and Laboratory Medicine Taipei Veterans General Hospital Taipei Taiwan; ^2^ Department of Otorhinolaryngology‐Head and Neck Surgery Taipei Veterans General Hospital Taipei Taiwan; ^3^ Department of Otorhinolaryngology, School of Medicine National Yang Ming Chiao Tung University Taipei Taiwan; ^4^ Department of Pathology, School of Medicine National Yang Ming Chiao Tung University Taipei Taiwan

**Keywords:** fine‐needle aspiration cytology, hypopharyngeal mass, pharyngoesophageal diverticulum, squamous cells, thyroid nodule, ultrasonography

## Abstract

Pharyngoesophageal diverticula (PED) are uncommon hypopharyngeal outpouchings that may mimic thyroid nodules because of their close anatomic proximity to the thyroid gland. We describe two cases in which PED presented as thyroid nodules and resulted in atypical cytological findings. The first case was a 56‐year‐old man, who presented with hoarseness and chronic cough. Ultrasonography showed a heterogeneous hypoechoic nodule, and fine‐needle aspiration (FNA) revealed benign squamous cells, food debris, inflammatory cells, and microorganisms, supporting the diagnosis of a PED. The second case was a 61‐year‐old man with chronic throat discomfort and an incidental neck mass initially suspected to be a benign thyroid nodule. Repeated FNA cytology consistently demonstrated benign squamous cells without thyroid follicular cells or colloid, and imaging correlation suggested a PED. These cases highlight the importance of correlating cytological and imaging findings when assessing presumed thyroid nodules to prevent misdiagnosis and unnecessary thyroid surgeries.

## 1 Introduction

Pharyngoesophageal diverticula (PED) are rare outpouchings of the hypopharyngeal mucosa protruding through areas of muscular weakness such as Killian's dehiscence [1, 2]. The overall incidence of PED is not well established in the literature. Among these lesions, Zenker's diverticulum, the most common subtype, has an estimated incidence of approximately 2.9 per 100,000 person‐years, shows a male predominance, and occurs with increasing frequency in individuals aged 70 years and older, whereas the rarer Killian–Jamieson diverticulum, Laimer's diverticulum, and pharyngoceles are only infrequently reported [2, 3, 4]. Typical symptoms of PED include dysphagia, regurgitation, and throat discomfort [2, 5]. Due to its anatomical proximity to the posterior aspect of the thyroid gland, PED may masquerade as a thyroid nodule on ultrasonography [6]. This can result in misdiagnosis and potentially unnecessary thyroid interventions. We report two cases initially suspected as thyroid nodules but ultimately diagnosed as PEDs on cytology and imaging.

## 2 Case Presentation

### 2.1 Case 1

**FIGURE 1:**
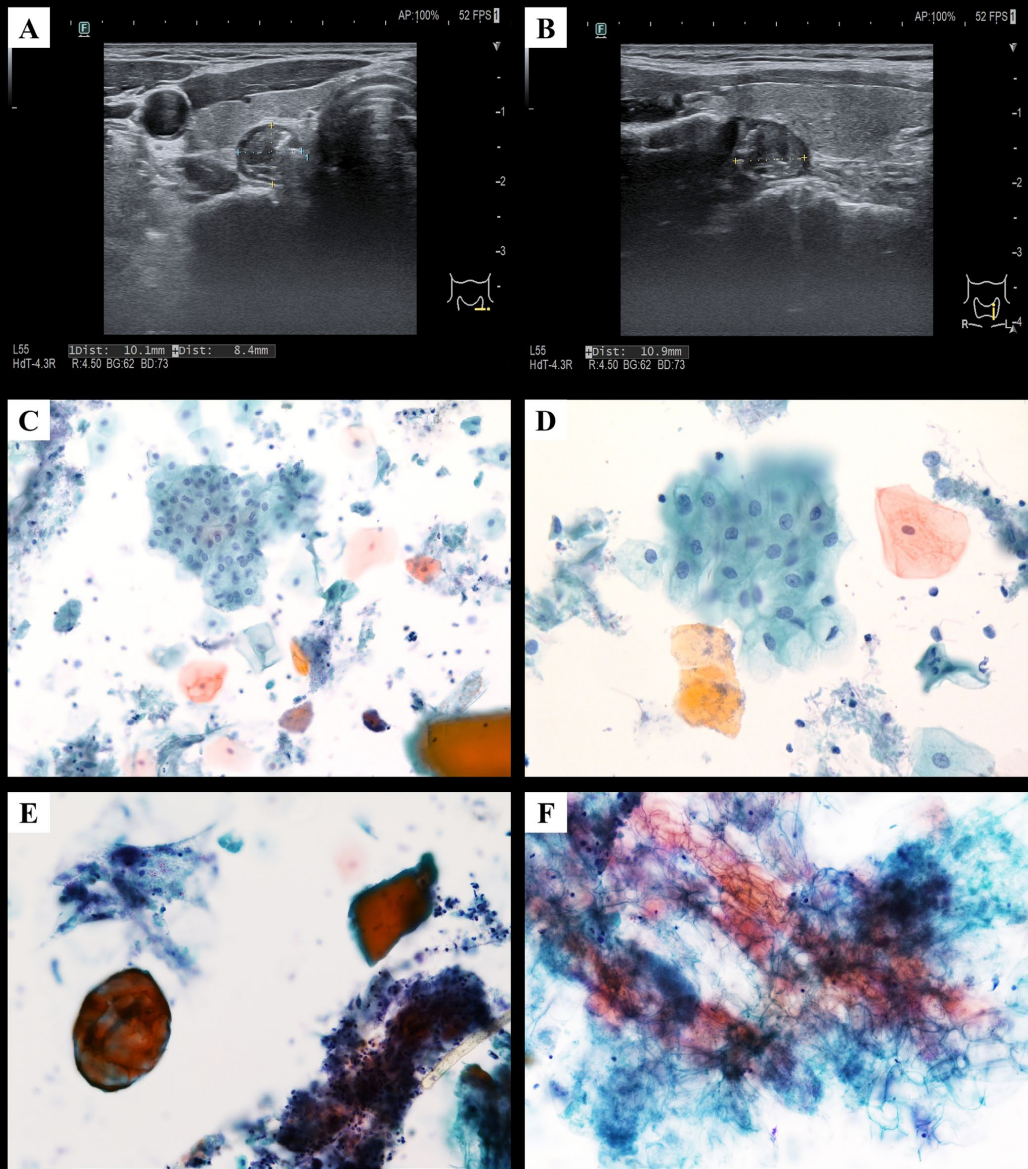
Case 1. (A) Transverse and (B) longitudinal ultrasound images of the left thyroid lobe showing a hypoechoic lesion with indistinct calcifications that appears to protrude from the thyroid gland. The LBC smear demonstrates: (C) a mixture of keratinized and non‐keratinized squamous cells admixed with food material, bacteria, and yeast (Papanicolaou stain, ×200); (D) abundant benign‐appearing squamous cells (Papanicolaou stain, ×400); (E) food debris (left) with a fragment of skeletal muscle (right), accompanied by inflammatory cells and yeast (Papanicolaou stain, ×400); and (F) vegetable‐type material with recognizable plant cell walls (Papanicolaou stain, ×400).

A 56‐year‐old man presented with progressive hoarseness and a dry, non‐productive cough for 3 months, which worsened with smoking. He had hyperlipidemia and a smoking history of 0.5 pack per day for 30 years. Laboratory evaluations, including thyroid‐stimulating hormone (TSH) and free thyroxine (fT4), were within normal limits. During a routine health checkup, ultrasonography incidentally revealed a left thyroid nodule with calcification. Repeat thyroid ultrasonography at our hospital showed a heterogeneous hypoechoic nodule in the left lobe, measuring 1.01 × 0.84 × 0.94 cm, with indistinct calcifications (Figure 1A,B). Ultrasound‐guided fine‐needle aspiration (FNA) yielded two conventional smears and one liquid‐based sample preserved in CytoRich Red fixative (Becton, Dickinson and Company, Franklin Lakes, NJ), which was processed into a liquid‐based cytology (LBC) smear using the SurePath method (Becton, Dickinson and Company). The procedure was well tolerated, and the patient was discharged stably. Cytological examination revealed benign squamous cells, food debris, and inflammatory cells accompanied by bacteria and yeast (Figure 1C–F). Based on the above findings, the final FNA diagnosis was benign. The differential diagnosis included cystic lesions with squamous cell metaplasia and PED. Correlating the benign cytology with food debris and sonographic findings, the lesion was suggestive of a PED. The patient was managed conservatively with close follow‐up, without immediate surgical intervention.

### 2.2 Case 2

A 61‐year‐old man presented with a longstanding burning and persistent foreign body sensation in the throat, which worsened in the supine position. A routine health examination conducted 13 years earlier incidentally revealed a right‐sided neck mass measuring 0.89 × 1.02 × 0.72 cm. Subsequent follow‐ups showed no significant interval growth, and a FNA at an outside hospital revealed benign cytology, leading to a presumptive diagnosis of a benign thyroid nodule.

**FIGURE 2:**
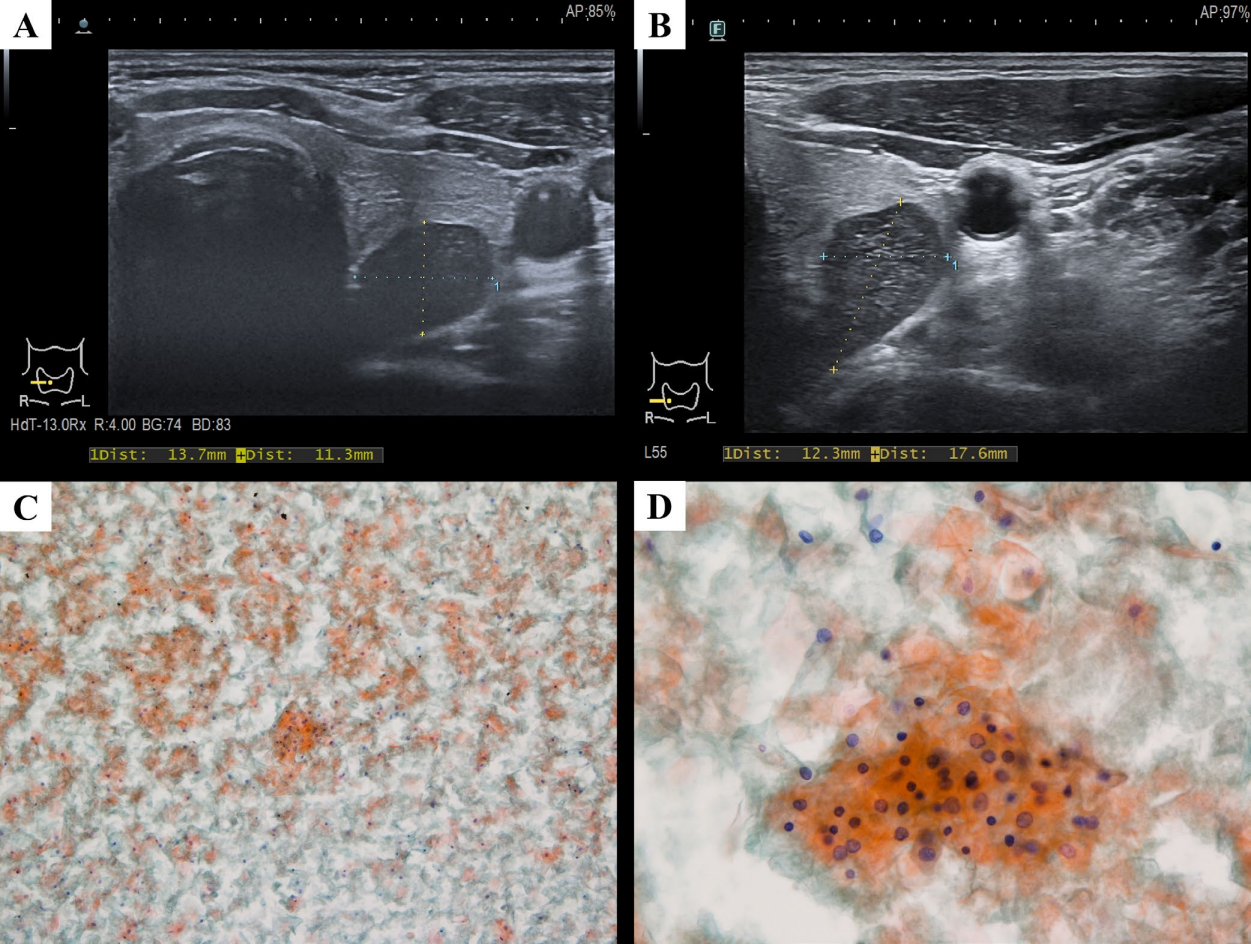
Case 2. (A) Transverse ultrasound image of the right thyroid lobe obtained 3 years earlier and (B) at the current presentation. The conventional smear from the prior examination shows: (C) abundant squamous cells (Papanicolaou stain, ×100) and (D) predominantly anucleated and scattered benign nucleated squamous cells (Papanicolaou stain, ×400).

Three years earlier, the patient presented to our hospital with persistent pharyngeal discomfort. Ultrasonography demonstrated a heterogeneous mass measuring 1.37 × 1.13 × 2.01 cm, located at the posterior aspect of the right thyroid lobe (Figure 2A). A parathyroid tumor was initially suspected by the clinician. Laboratory evaluation, including serum calcium, intact parathyroid hormone, TSH, and fT4, yielded results within normal limits. Ultrasound‐guided FNA of the lesion was performed uneventfully, and cytological examination revealed only benign squamous cells without evidence of malignancy (Figure 2C,D). The clinician recommended a right thyroid lobectomy; however, the patient was subsequently lost to follow‐up for 3 years until now.

A repeat thyroid ultrasound identified an anechoic nodule in the right thyroid region, measuring 1.23 × 1.76 × 2.18 cm (Figure 2B), showing no significant interval change in size. Laboratory results remained unremarkable. A repeat ultrasound‐guided FNA was performed uneventfully without complications, and cytological evaluation again demonstrated numerous benign squamous cells, with no food material or inflammatory debris identified. Based on these findings, the final FNA diagnosis was benign and the differential diagnosis mainly included benign squamous‐lined cyst and squamous metaplasia. In addition, a computed tomography scan was performed which showed the lesion to be closely related to the junction between the pharynx and the esophagus. Given the similar FNA results and the stable size of the lesion in an interval of 3 years, the lesion was suggestive of a PED. The patient was managed conservatively with close follow‐up, without immediate surgical intervention.

## 3 Discussion

We present two cases of PEDs initially suspected to be thyroid nodules, located on the left and right sides, respectively. Previous reports of PEDs mimicking thyroid nodules have been documented, predominantly involving the left thyroid region, whereas right‐sided occurrences remain rare [4, 6, 7, 8, 9, 10, 11]. The etiology of PED is multifactorial but largely attributed to cricopharyngeal dysfunction, leading to mucosal herniation through Killian's dehiscence [1, 2, 3, 5]. Previous studies have shown that FNA cytology of PED typically reveals abundant benign squamous epithelium together with inflammatory cells, microorganisms, necrotic debris, and food particles [4, 6, 7, 8, 9, 10, 11]. The first case demonstrated typical cytological features of a PED, particularly squamous cells admixed with food debris, whereas the second case revealed only benign squamous cells. We propose that this discrepancy may be explained by an anatomic variant wherein the diverticulum forms a blind‐ending pouch or maintains only a narrow communication, resulting in an alternative cytological pattern of PED on FNA cytology.

Regarding the cytological findings, squamous cells are uncommonly identified in thyroid aspirates. When present, they may originate from thyroid lesions ranging from benign squamous metaplasia secondary to chronic inflammation to malignant entities, including papillary thyroid carcinoma (PTC) with squamous differentiation, anaplastic carcinoma, or mucoepidermoid carcinoma [4, 12]. Alternatively, the presence of squamous cells should also raise consideration of other non‐thyroid lesions such as PEDs, lymphoepithelial cysts, epidermoid cysts, or thyroglossal duct cysts. In our cases, the integration of the cytological findings with the sonographic correlation favored non‐thyroidal origin [4, 12]. Considering the rarity of PED and the potential diagnostic pitfalls in thyroid FNA, a diagnosis was made only when benign cytological features were concordant with sonographic findings in both cases.

In our cases, the lesions appeared as well‐defined nodules closely related to the thyroid on ultrasonography, making it difficult to rule out the possibility of thyroid tumors. Thyroid malignancies, particularly PTC, typically appear on ultrasonography as hypoechoic nodules and microcalcifications, which may complicate the accurate interpretation of imaging findings in cases of PEDs [12, 13]. When only thyroid ultrasonography is available and FNA is not performed, additional imaging studies such as a barium swallow study or esophagography are essential for the definitive diagnosis of a PED [1, 3, 6]. Given the cytologic and sonographic findings and the absence of urgent indications, esophagography was not performed in either patient.

In conclusion, our report underscores the clinical importance of correlating FNA cytology results with clinical and imaging findings, highlighting the need for a comprehensive diagnostic approach to avoid unnecessary surgical interventions and improve patient safety.

## Author Contributions

Yu‐Wei Lin performed a literature search and drafted the manuscript. Tzu‐Hang Kao and Tsung‐Lun Lee provided patient acquisition. Jen‐Fan Hang conceived the manuscript design and edited the manuscript. All authors reviewed and approved the final manuscript.

## Funding

The authors have nothing to report.

## Conflicts of Interest

The authors declare no conflicts of interest.

## Data Availability

Data sharing not applicable to this article as no datasets were generated or analyzed during the current study.
